# Synthesis of Three-Dimensional Carbon Nanostructure/Copper Nanowire for Additive Interface Layer of Ionic Polymer Metal Composite

**DOI:** 10.3390/nano10030423

**Published:** 2020-02-28

**Authors:** Seongjun Park, Minjeong Park, Seonpil Kim, Minhyon Jeon

**Affiliations:** 1 Department of Nanoscience and Engineering, Center for Nano Manufacturing, Inje University, Gimhae 50834, Korea; power0092@gmail.com (S.P.); mjpark9121@gmail.com (M.P.); 2 Department of Military Information Science, Gyeongju University, Gyeongju 38065, Korea; seonpil@gu.ac.kr

**Keywords:** carbon nanotube, graphene, Cu-NW, three-dimensional carbon nanostructure, ionic polymer metal composite, additive layer

## Abstract

Additive interface materials for improved ionic polymer metal composite (IPMC) actuator performance are being investigated. In this study, three-dimensional carbon nanostructure/copper nanowire (3DC Cu-NW) with a novel structure was synthesized via low-pressure chemical vapor deposition. An IPMC actuator with a 3DC Cu-NW interface layer was fabricated, which exhibited improved actuation performance, long-term stability, and electrochemical properties. The proposed 3DC consists of carbon nanotubes (CNTs) and graphene, grown using an Fe catalyst and CH_4_ gas, respectively. We optimized the growth conditions (Fe catalyst: 12.5 mg/L, CH_4_: 20 sccm) to achieve a 3DC with an appropriate thickness and a large specific surface area. The 3DC Cu-NW benefited from a Cu oxidation prevention property and a large specific surface area. The electrochemical properties and actuation performance of the IPMC actuator improved with an increased 3DC Cu-NW concentration. An IPMC actuator with a 0.6 wt% 3DC Cu-NW interface layer exhibited 1.3- and 5.6-fold electrochemical property and actuation performance improvement, respectively, over an IPMC actuator with no 3DC Cu-NW interface layer. These results show that the proposed 3DC Cu-NW has potential as an IPMC actuator interface material, and that 3DC Cu-NW synthesis and application technology can be applied to future research on sensor, actuator, and flexible devices.

## 1. Introduction

Compared to traditional machine actuators, smart actuators have advantages such as miniaturization and simplification of structure. Piezoelectrics, ferroelectrics, shape memory alloys and electroactive polymers were activated by transforming electrical energy into mechanical energy [1,2,3,4] and MoS_2_, graphene, and graphene nanoplatelet-based polymer actuators have a mechanism that operates by transforming light energy into mechanical energy [5,6,7]. Among them, ionic polymer metal composite (IPMC) has been studied as an actuator for application in the biomedical, industrial, and medical fields, because of its advantages of low mass, flexibility, and its large bending deformation with low driving voltage [8,9,10,11]. IPMC consists of electrodes and an ionic polymer membrane. When the input voltage is supplied to IPMC electrodes, the hydrated protons in the ionic polymer is moved in cathode. Cathode with a relatively high proton concentration expand and the anode is compressed [4,5] so the bending motion of the IPMC actuator can occur. If an alternating current voltage is provided, the above phenomenon repeats when voltage is applied. Recently, the interface between the electrode and ionic polymer membrane has attracted research attention, with regard to enhancing the actuation performance, long-term stability, and electrochemical properties of the IPMC actuator, for potential application in sensor and actuator devices. In particular, many researchers [12,13,14,15,16,17,18] have investigated various additives for Nafion, which is widely used in ion exchange polymers and interface layers because of features such as its chemical stability, commercial availability, thermal stability, and high proton conductivity. Metallic materials such as Pt, Au, and TiO_2_ nanoparticles, as well as carbon materials such as carbon nanotubes (CNTs) and graphene, have been used as additives [12,13,14,15,16,17,18]. However, improvements to the actuation performance, long-term stability, and electrochemical properties of the IPMC actuator are still required. Non-oxidative materials with large specific surface areas, nanowire, or nanotube structures can potentially facilitate these improvements.

Among the various nanowire materials, copper nanowire (Cu-NW) has the advantage of high electrical conductivity, superior structural flexibility, and low-cost synthesis [19]. Furthermore, Cu-NW can constitute an alternative metal nanoparticle because of its very large or easily tunable aspect ratio and specific surface area [20,21]. However, Cu-NW can have problems with electron transport and conductivity because of its low chemical stability and easy oxidation. This problem is solved by growing graphene on the Cu-NW surface. Graphene is a smart nanomaterial with excellent electrical conductivity, electrochemical stability, and gas-impermeability [22,23,24,25]. When graphene-based composites are used in Nafion, the mechanical and electrochemical properties are enhanced, and when this material is applied to an IPMC actuator, the actuation performance is improved. Importantly, the electrochemical properties (such as the capacitance) of the graphene/CNT structure are reported to be superior to those of graphene alone [26]. Additionally, because CNTs have nanotube structures, large specific surface areas, various surface functional groups, and high strength, they can enhance the actuation performance, as well as the electrochemical properties of an IPMC actuator containing a graphene/CNT structure.

In this study, CNTs and graphene are grown on Cu-NW using an Fe catalyst and CH_4_ gas in a low-pressure chemical vapor deposition (LP-CVD) system. We successfully synthesize three-dimensional carbon nanostructure/copper nanowire (3DC Cu-NW) with a new structure. Notably, application of the CNT/graphene onto the Cu-NW prevents Cu oxidation. We then fabricate an IPMC actuator with a 3DC Cu-NW interface layer using the hot-press method. The electrochemical properties of the IPMC actuator with the 3DC Cu-NW interface layer are increased by 3DC Cu-NW with a large specific surface area, and its actuation performance is enhanced with increased 3DC Cu-NW concentration.

## 2. Materials and Methods

### 2.1. Materials

Copper nitrate hydrate (99.999% Cu(NO_3_)_2_), sodium hydroxide (10.0 N standardized solution, NaOH), sodium hydroxide (pearl, 97% NaOH), and Cu foil (thickness: 0.025 mm, 99.999%) were purchased from Sigma Aldrich, Saint Louis, MO, USA. Hydrazine solution (35 wt% in H_2_O, N_2_H_4_) was purchased from Alfa Aesar, Heverville, MA, USA. These materials were used for the Cu-NW synthesis. Iron (Fe) nanoparticles (99.5+%, 30 nm, Fe_2_O_3_ alpha) were used as a catalyst for the CNT growth, and were purchased from RND Korea, Ltd, Gwangmyeong, Korea. A silver nanowire (Ag-NW) solution was purchased from Duksan Hi-Metal (Ulsan, Korea), and graphene oxide (GO) was purchased from Grapheneall, Ltd, Siheung, Korea. Nafion-117 and 20 wt% Nafion solution were purchased from the DuPont Company (Midland, MI, US), and were used as the ionic polymer membrane of the IPMC and the interface material with the Cu-NW, respectively. The cation used for the actuation of the IPMC in the Nafion was 1-Ethyl-3 methylimidazolium trifluoromethylsulfonate (EMIM-Otf, ionic liquid), and this was purchased from Merck KGaA (Darmstadt, Germany). Polyvinylidene difluoride (PVDF) membrane filter paper (pore size: 0.20 μm, diameter: ϕ47 mm) was purchased from Hyundai Micro., Ltd, Seongnam, Korea, and was used for Cu-NW synthesis and fabrication of the GO/Ag-NW paper electrode.

### 2.2. Growth of the Three-Dimensional Carbon Nanostructure on Copper Nanowire (3DC Cu-NW)

In the typical Cu-NW synthesis process [20,21], the NaOH of 15 M and Cu(NO_3_)_2_ of 0.1 M were prepared before Cu-NW synthesis, using a deionized water base solution. Then, 20 mL NaOH, 1 mL Cu(NO_3_)_2_, and 100 μL ethylenediamine (EDA) were mixed in a round flask in a 60 °C water bath. The solution was stirred at 700 RPM for 3 min. After stirring, 20 to 45 μL N_2_H_4_ was added to the solution. The stirring stopped after 2 min. After 1 h, the Cu-NW floated on the surface of the solution, and its color changed from colorless to dark red. The Cu-NW was rinsed in deionized water and harvested on a polyethersulfone (PES) membrane with a filtration process. Herein, the EDA demonstrated an anisotropic growth of Cu-NW [20,21,27]. The anisotropic-grown Cu-NW was easily obtained on the PES membrane filter paper.

Graphene/Cu-NW (G/Cu-NW) was grown using LP-CVD. The Cu-NW was placed in a tungsten (W) boat, and the W boat was wrapped in Cu foil. The Cu-NW was annealed in an atmosphere of 200 sccm argon (Ar) and 50 sccm H_2_ at 350 °C with LP-CVD over a 15 min period. In the graphene growth step, the temperature gradient consisted of a 900 °C heating zone and a 650 °C growth zone. Graphene was also grown in 10 to 60 sccm CH_4_ for 10 min. After the graphene growth process was completed, the quartz was cooled to room temperature. 

Fe nanoparticles were used as a catalyst for CNT growth on the G/Cu-NW. Fe nanoparticles at a 7.5 to 17.5 mg/L_ethanol_ concentration were dispersed using a probe sonicator for 10 min. The G/Cu-NW was added to the suspension and dispersed using a bath sonicator for 1 min. After stirring for 1 h, the Fe catalysis on the G/Cu-NW (Fe/G/Cu-NW) was harvested with a vacuum oven (50 °C, over 1 h). The CNTs were grown using LP-CVD. To remove organics, the Fe/G/Cu-NW composite was annealed at 300 °C in 1000 sccm Ar, and 100 sccm dihydrogen (H_2_) for 10 min. The temperature gradient consisted of a 700 °C heating zone and 540 °C growth zone in the CNT growth step. CNTs were also grown in 2 sccm acetylene for 12 min. After CNT growth, the specimens were cooled to room temperature. Hence, the 3DC Cu-NW with the new structure was successfully fabricated.

### 2.3. Fabrication of the IPMC Actuator based on 3DC Cu-NW

A GO/Ag-NW paper electrode was prepared according to the method of Yoo et al. [28]. The Nafion ion exchange process was performed following a previously reported method [29]. The 3DC Cu-NW was stirred in Nafion solution and dispersed using a bath sonicator for 10 min. The 3DC Cu-NW/Nafion composite specimens were prepared with 0.2, 0.4, and 0.6 wt% 3DC Cu-NW content. The 3DC Cu-NW/Nafion composite was painted directly on the Nafion membrane. To obtain a 3DC Cu-NW interface layer between the GO/Ag-NW electrode and Nafion membrane, the GO/Ag-NW electrode was placed on both sides of the 3DC Cu-NW/Nafion composite painted on the Nafion membrane. Attachment was achieved using a hot press at 0.1 MPa and 100 °C for 2.5 min. The filter paper was removed using acetone solution. Finally, the IPMC actuator with the 3DC Cu-NW interface layer was cut to dimensions of 0.5 × 4.0 cm^2^.

### 2.4. Characteristics

**Figure 1:**
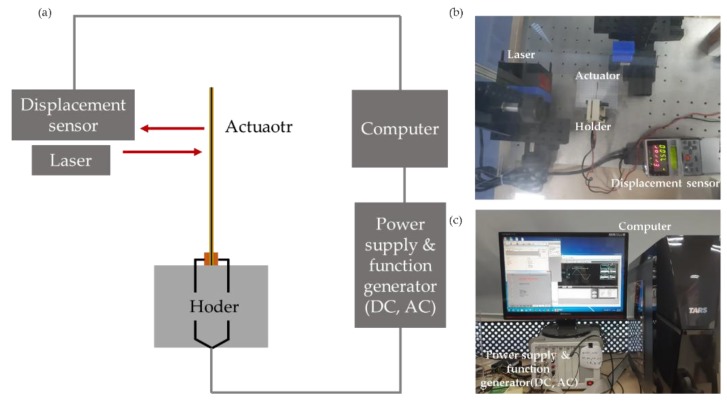
(**a**) Schematic diagram, and (**b**,**c**) photographs of actuation measurement system.

The 3DC Cu-NW morphology was investigated using field-emission scanning electron microscopy (FE-SEM; S-4300, Hitachi, Tokyo, Japan). The actuation performance of the IPMC actuator was measured using a laser displacement sensor (ZS-LD80, OMRON Korea, Seoul, Korea). Figure 1 showed the mechanical property measurement system with the laser displacement sensor of the IPMC actuator. This system was made by ourselves. The functional groups of the 3DC Cu-NW were observed using Fourier-transform infrared spectroscopy (FT-IR; FT/IR-6300, JASCO, Tokyo, Japan). The electrochemical properties of the IPMC actuator were measured using a cyclic voltammetry system (CompactStat.h10800, Ivium, Eindhoven, Netherlands). The oxidation of the Cu-NW and G/Cu-NW was observed using an X-Ray diffractometer (XRD; Ultima IV, Rigaku, Tokyo, Japan).

## 3. Results

### 3.1. Characteristics of 3DC Cu-NW

**Figure 2:**
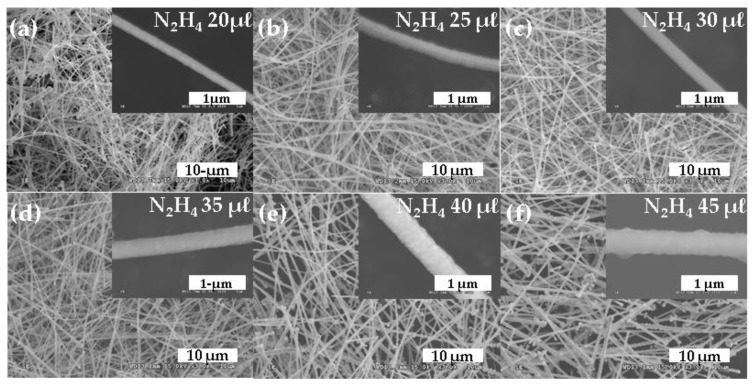
SEM images of Cu-NW for N_2_H_4_ concentrations of (**a**) 20, (**b**) 25, (**c**) 30, (**d**) 35, (**e**) 40, and (**f**) 45 μL.

To synthesize Cu-NWs with various diameters, we controlled the N_2_H_4_ concentration. Figure 2 shows various Cu-NW morphologies according to the N_2_H_4_ concentration; the inset images show parts of individual Cu-NWs. The average Cu-NW diameter increased from 183 to 443 nm, in accordance with increases in the N_2_H_4_ concentration from 20 to 45 μL. Particle-like surfaces were observed for the Cu-NW specimens. In particular, the Cu-NW shape was not maintained for the 45 μL N_2_H_4_ concentration. For Cu-NW shape maintenance under graphene growth temperatures, Cu-NW diameters exceeding 350 nm are needed. Thus, a Cu-NW specimen with a less particle-like surface, and an approximate diameter of 362 nm, was selected for use in the graphene growth process on the Cu-NW surface; this Cu-NW was obtained using 40 μL N_2_H_4_ concentration.

**Figure 3:**
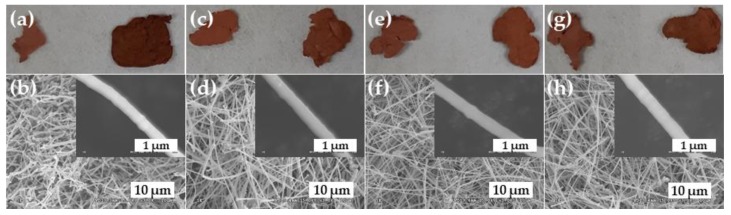
(**a**,**c**,**e**,**g**) Optical images of G/Cu-NW specimens before and after oxidation (specimens on left and right of each image, respectively), and (**b**,**d**,**f**,**h**) SEM images of G/Cu-NWs for CH_4_ concentrations of (**a**,**b**) 10, (**c**,**d**) 20, (**e**,**f**) 30, and (**g**,**h**) 40 sccm.

Graphene was grown on the Cu-NW using the methods of Xu et al. [24] and Park et al. [27]. These two methods were used to protect the Cu-NW shape from the vapor pressure and heat treatment. Figure 3 shows the graphene growth achieved according to various CH_4_ concentrations. The various G/Cu-NWs were not melted, and their surfaces were smooth with no particle-like Cu-NW surfaces as a result of the H_2_ treatment and annealing process. Furthermore, all the G/Cu-NWs had similar shapes regardless of the changing CH_4_ concentrations (Figure 3b–h and insets). We oxidized all the G/Cu-NWs under 80 °C for 80 h, so as to observe the graphene step coverage and gas impermeability. Figure 3a,c,e and g are optical images of the Cu-NW specimens before and after oxidation (the specimens on the left and right of each image, respectively), for CH_4_ concentrations of 10, 20, 30, and 40 sccm, respectively. The G/Cu-NW obtained using 10 sccm CH_4_ changed color significantly from brown to dark brown, indicating poor step coverage of the graphene on the Cu-NW. In contrast, the G/Cu-NWs prepared using the other CH_4_ concentrations had even and similar colors after the oxidation process. Thus, good graphene step coverage was achieved for the G/Cu-NW specimens prepared with CH_4_ content of 20 sccm or more, and the Cu-NW was not oxidized.

**Figure 4:**
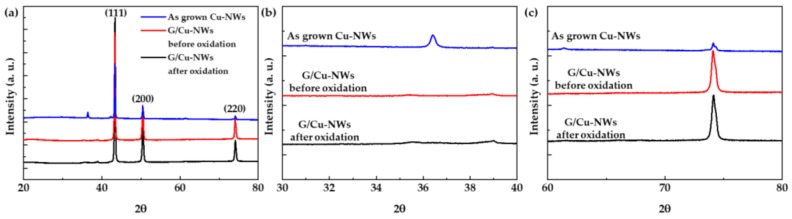
XRD spectra of as-grown Cu-NW and 20 sccm-CH_4_ G/Cu-NW before and after oxidation; (**a**) full XRD spectra, (**b**) Cu_2_O and CuO peaks, and (**c**) CuO peaks.

The oxidation of the as-grown Cu-NW and 20 sccm CH_4_ G/Cu-NW samples (before and after oxidation) could be determined from the XRD spectra (Figure 4). Figure 4a shows the total XRD data of the samples from 20 to 80 degrees. The general Cu peaks of (111), (200), and (220) are apparent at approximately 44, 52, and 76 degrees, respectively. The G/Cu-NW had higher Cu intensity than the as-grown Cu-NW, indicating that the former had higher crystallinity and that the natural oxidation groups of the Cu-NW were removed by the graphene growth. Figure 3b,c show the cuprous oxide (Cu_2_O) and cupric oxide (CuO) peaks of the samples. For the as-grown Cu-NW, the Cu_2_O peaks were observed at approximately 36 and 42 degrees, and the CuO peaks were observed at approximately 39, 61, and 74 degrees. However, Cu_2_O and CuO peaks were not observed for the G/Cu-NW before or after the oxidation process. These results agree with Figure 3, in that they indicate good graphene step coverage without oxidation of the Cu-NW in the G/Cu-NW prepared using 20 sccm CH_4_. These results also indicate that the graphene prevented Cu oxidation. 

**Figure 5:**
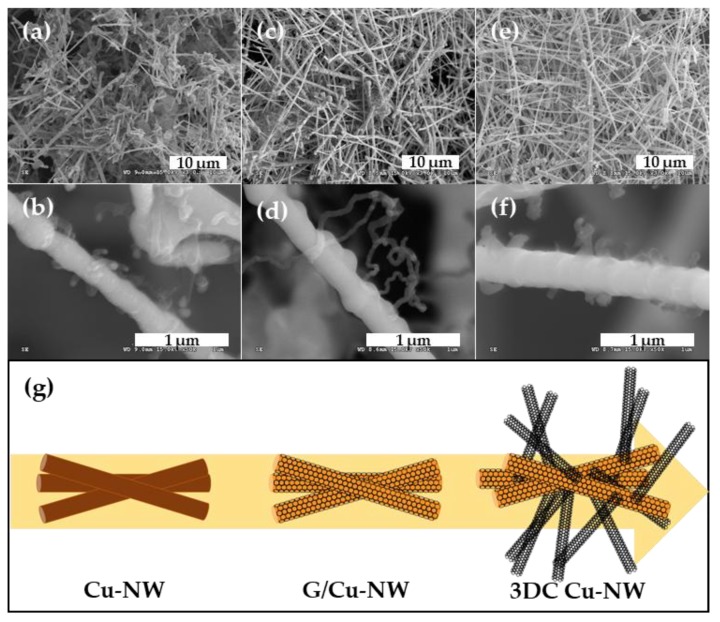
SEM images of carbon nanotubes (CNT) growth for Fe catalyst concentrations of (**a**,**b**) 7.5, (**c**,**d**) 12.5, and (**e**,**f**) 17.5 mg/L; (**g**) Schematic diagram of structural changes from Cu-NW to 3DC Cu-NW.

CNTs were grown on the synthesized G/Cu-NW prepared under 20 sccm CH_4_ using various Fe catalyst concentrations. Figure 5 shows the 3DC Cu-NWs grown using Fe catalyst concentrations of 7.5, 12.5, and 17.5 mg/L and LP-CVD. The CNTs grown with the 7.5 mg/L Fe catalyst were short (Figure 4a,b), while the CNTs were improperly grown for the 17.5 mg/L Fe catalyst (Figure 5e,f). However, the CNTs were well grown with the 12.5 mg/L Fe catalyst concentration (Figure 5c,d); thus, the 12.5 mg/L was an appropriate Fe concentration for the CNT growth on the prepared Cu-NW. Furthermore, the largest specific surface area was obtained for the CNT grown using the 12.5 mg/L Fe catalyst. It was therefore apparent that the 3DC Cu-NW grown using the 12.5 mg/L Fe catalyst could potentially be used to enhance the electrochemical properties of the IPMC. Figure 5g schematically illustrates the growth process from Cu-NW to 3DC Cu-NW, according to the corresponding structures.

**Figure 6:**
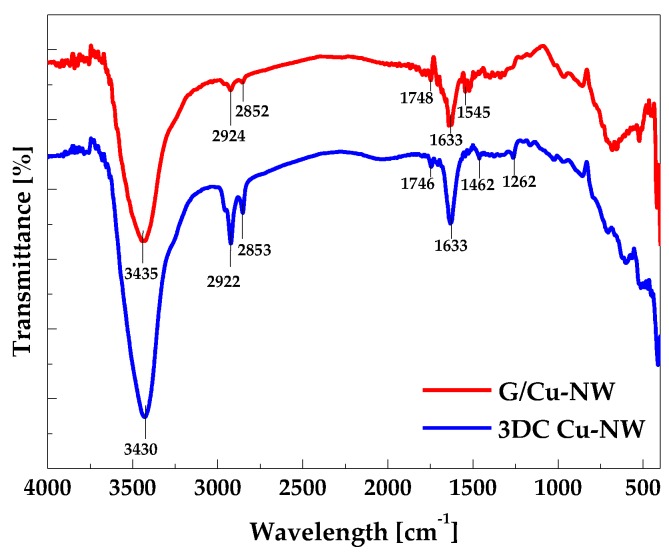
FT-IR spectra of G/Cu-NW and 3DC Cu-NW.

Figure 6 shows the FT-IR spectra of the G/Cu-NW and 3DC Cu-NW prepared using the 12.5 mg/μL Fe catalyst. For both specimen types, peaks corresponding to the O–H stretching vibration, C–H asymmetric and symmetric CH_2_ stretching vibration, C=O stretching vibration, and C=C skeletal vibration of graphitic domains were observed at approximately 3430 and 3435, from 2852 to 2924, 1746 and 1748, 1633 cm^−1^, respectively [30,31,32,33]. N–H bend vibration (at 1545 cm^−1^) was only observed at G/Cu-NWs. Note that the N–H group appeared because EDA with an amine group was used in the Cu-NW synthesis. After the CNTs were grown on the G/Cu-NW, the intensities of the carbon groups increased, and the intensity of N–H bend vibration reduced because of CNT growth on G/Cu-NW [34,35]. Also, C–O stretching vibration (at 1462 cm^−1^) and C–O stretching vibration (1262 cm^−1^) were created. This means that the 3DC Cu-NW had many functional groups on its surface compared to the G/Cu-NW. Note that various graphene and CNT functional groups help improve the electrical and chemical properties of the material.

### 3.2. Characteristics of the IPMC Actuator Based on 3DC Cu-NW 

**Figure 7:**
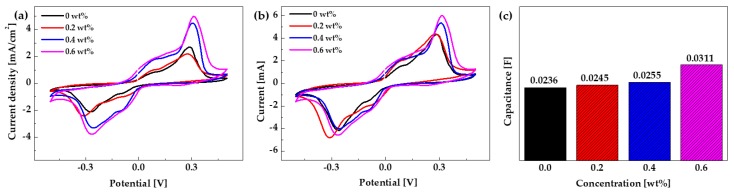
(**a**) Current density and (**b**) *I*–*V* curves, and (**c**) capacitance of IPMC actuator according to 3DC Cu-NW concentration.

An IPMC actuator based on a GO/Ag-NW electrode with an interface layer was fabricated. The 3DC Cu-NW prepared using 12.5 mg/L Fe catalyst was used as the IPMC interface layer between the Nafion and the electrode. To observe the electrochemical properties of the IPMC actuator, 0.2, 0.4, and 0.6 wt% 3DC Cu-NW samples were used as interface layers. The electrochemical properties of the IPMC actuator were measured under ±0.5 V, at a scan rate of 50 mV/s, and current range of 10 mA in LiCl solution. Figure 7 shows the electrochemical properties recorded for actuators with 0 to 0.6 wt% 3DC Cu-NW content. Figure 7a shows the current density of the IPMC actuator, which increased with increasing 3DC Cu-NW concentration. Figure 7b shows the current–voltage (*I*–*V*) curve of the IPMC actuator. The area of the *I*–*V* curve indicates the capacitance of the IPMC actuator; these results are shown in Figure 7c. From these graphs, it is apparent that the IPMC actuator with 0.6 wt% 3DC Cu-NW had superior electrochemical properties to the other specimens. Importantly, this result means that the IPMC actuator with the 3DC Cu-NW interface layer had a greater charge storage than that with no 3DC Cu-NW interface layer.

**Figure 8:**
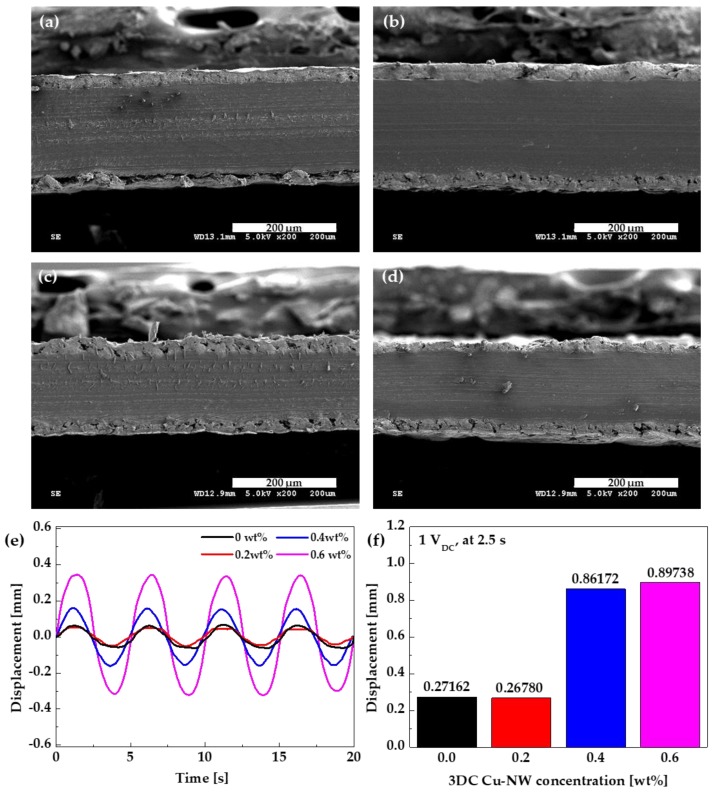
Cross sections of IPMC actuators with (**a**) 0, (**b**) 0.2, (**c**) 0.4, and (**d**) 0.6 wt% 3DC Cu-NW; (**e**) Displacement vs. time (±1 V_AC_, 0.2 Hz), and (**f**) real-time displacement at 2.5 s under 1 V_DC_ results for IPMC actuators according to 3DC Cu-NW concentration.

Figure 8a–d show the cross sections of each IPMC actuator according to the 3DC Cu-NW concentration. The Nafion membrane and electrode were well attached in all IPMC actuators with a 3DC Cu-NW interface layer. However, poor attachment of these components was observed for the IPMC actuator without the 3DC Cu-NW interface layer. To observe the actuation characteristics, an AC voltage was injected into the IPMC actuators with 3DC Cu-NW. The DC voltage was 0 to 2.5 s at 1 V_DC_. The actuation performances of the IPMC actuators with different 3DC Cu-NW concentrations are shown in Figure 8e,f. Figure 8e shows the displacement performance according to time under 1 V_AC_ and 0.2 Hz. There were notable increases in displacement for the IPMC actuators with more than 0.4 wt% 3DC Cu-NW, with the largest displacement being obtained for the IPMC actuator with 0.6 wt% 3DC Cu-NW. Figure 8f shows the real-time displacement values for the IPMC actuators at 2.5 s after 1 V_DC_ input. The displacement of the IPMC actuator with 0.6 wt% 3DC Cu-NW was larger than those of the others at 2.5 s; thus, this actuator had a higher response rate. Hence, we observed that the newly fabricated 3DC Cu-NW had the potential to enhance the electrochemical properties and actuation performance of the IPMC actuator, with 0.6 wt% 3DC Cu-NW content giving the best results.

## 4. Conclusions

3DC Cu-NW with a new structure was synthesized in this study and employed as an interface layer in an IPMC actuator. The 3DC Cu-NW used in this device had an average diameter of approximately 362 nm. We successfully synthesized 3DC Cu-NWs with different properties according to their CH_4_ and Fe catalyst concentrations by using an LP-CVD system to prevent oxidation of the Cu-NW. The Cu-NW, G/Cu-NW, and 3DC Cu-NW growth steps were each optimized by considering SEM and FT-IR results. The morphology of the 3DC Cu-NW was well preserved, and it was confirmed that Cu oxidation did not occur. The fabricated IPMC actuator with a 3DC Cu-NW interface layer varied according to the 3DC Cu-NW concentration. The current density and capacitance were increased according to the added 3DC Cu-NW, which had a large specific surface area. In particular, the capacitance, displacement, and response rate of the IPMC actuator with 0.6 wt% 3DC Cu-NW were 1.3, 5.6, and 3.3 times higher than those of an IPMC actuator with no 3DC Cu-NW, respectively. Thus, the fabricated 3DC Cu-NW has potential as an interface material for an IPMC actuator. Furthermore, the synthesis technology used to prepare the newly structured 3DC Cu-NW with a large specific surface area could promote applications involving carbon-based devices, flexible actuators, and electrochemical sensors.
